# Longitudinal analysis of treatment-induced genomic alterations in gliomas

**DOI:** 10.1186/s13073-017-0401-9

**Published:** 2017-02-02

**Authors:** E. Zeynep Erson-Omay, Octavian Henegariu, S. Bülent Omay, Akdes Serin Harmancı, Mark W. Youngblood, Ketu Mishra-Gorur, Jie Li, Koray Özduman, Geneive Carrión-Grant, Victoria E. Clark, Caner Çağlar, Mehmet Bakırcıoğlu, M. Necmettin Pamir, Viviane Tabar, Alexander O. Vortmeyer, Kaya Bilguvar, Katsuhito Yasuno, Lisa M. DeAngelis, Joachim M. Baehring, Jennifer Moliterno, Murat Günel

**Affiliations:** 1 Yale Program in Brain Tumor Research, Yale School of Medicine, New Haven, CT USA; 2 Department of Neurosurgery, Yale School of Medicine, New Haven, CT USA; 3 Department of Genetics, Yale School of Medicine, New Haven, CT USA; 4 Department of Neurobiology, Yale School of Medicine, New Haven, CT USA; 5 Yale Program on Neurogenetics, Yale School of Medicine, New Haven, CT USA; 6 Department of Pathology, Yale School of Medicine, New Haven, CT USA; 7 Department of Neurosurgery, Acıbadem University School of Medicine, Istanbul, Turkey; 8 Department of Neurosurgery, Memorial Sloan Kettering Cancer Center, New York, NY USA; 9 Department of Neurology, Memorial Sloan Kettering Cancer Center, New York, NY USA; 10 Yale Center for Genome Analysis, Yale School of Medicine, Orange, CT USA; 11 Department of Neurology, Yale School of Medicine, New Haven, CT USA; 12 Yale Brain Tumor Center, Yale School of Medicine, New Haven, CT USA; 13 Yale Comprehensive Cancer Center, Yale School of Medicine, New Haven, CT USA; 14 Yale Neurosurgery, PO Box 208082, New Haven, CT 06520-8082 USA

**Keywords:** Genomics-guided precision medicine, Tumor evolution, Longitudinal genomic analysis, Immune checkpoint inhibition, Mismatch repair deficiency, Glioma

## Abstract

**Background:**

Glioblastoma multiforme (GBM) constitutes nearly half of all malignant brain tumors and has a median survival of 15 months. The standard treatment for these lesions includes maximal resection, radiotherapy, and chemotherapy; however, individual tumors display immense variability in their response to these approaches. Genomic techniques such as whole-exome sequencing (WES) provide an opportunity to understand the molecular basis of this variability.

**Methods:**

Here, we report WES-guided treatment of a patient with a primary GBM and two subsequent recurrences, demonstrating the dynamic nature of treatment-induced molecular changes and their implications for clinical decision-making. We also analyze the Yale-Glioma cohort, composed of 110 whole exome- or whole genome-sequenced tumor-normal pairs, to assess the frequency of genomic events found in the presented case.

**Results:**

Our longitudinal analysis revealed how the genomic profile evolved under the pressure of therapy. Specifically targeted approaches eradicated treatment-sensitive clones while enriching for resistant ones, generated due to chromothripsis, which we show to be a frequent event in GBMs based on our extended analysis of 110 gliomas in the Yale-Glioma cohort. Despite chromothripsis and the later acquired mismatch-repair deficiency, genomics-guided personalized treatment extended survival to over 5 years. Interestingly, the case displayed a favorable response to immune checkpoint inhibition after acquiring mismatch repair deficiency.

**Conclusions:**

Our study demonstrates the importance of longitudinal genomic profiling to adjust to the dynamic nature of treatment-induced molecular changes to improve the outcomes of precision therapies.

**Electronic supplementary material:**

The online version of this article (doi:10.1186/s13073-017-0401-9) contains supplementary material, which is available to authorized users.

## Background

**Fig. 1:**
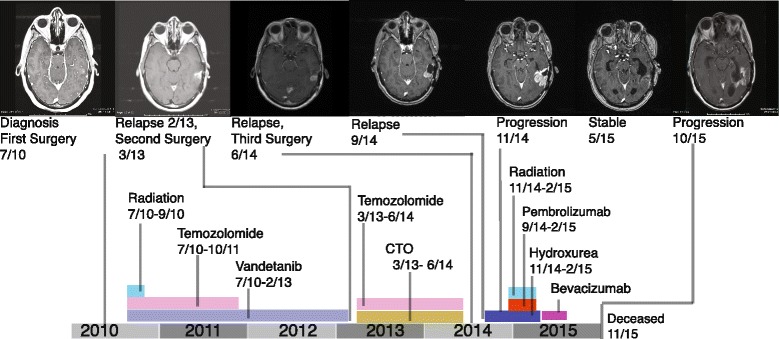
Clinical progression of the presented case. Treatments are represented with *colored bars*: temozolomide treatment in *pink*; vandetanib (targeted EGFR treatment) in *purple*, carboxyamidotriazole orotate (*CTO*; targeted phosphoinositide 3-kinase treatment) in *yellow*, immunotherapy in *dark blue*, hydroxurea in *red*, radiation in *light blue*, and bevacizumab in *magenta*

Glioblastoma multiforme (GBM) constitutes 15.6% of all and 45.2% of malignant brain tumors, with a poor prognosis and 5-year survival in less than 5% of cases. Besides the heterogeneity among different histologies and grades, glial tumors also show significant inter- and intra-tumor heterogeneity [1, 2], a feature that carries important implications for both targeted and standard-of-care treatments. Here, we present longitudinal whole-exome sequencing (WES) of a GBM patient undergoing treatment and report rapid evolution in response to targeted clinical approaches. Our longitudinal analysis spanned 5 years and revealed how the genomic profile evolved under the pressure of targeted therapy, specifically leading to the eradication of treatment-sensitive clones while enriching for those that showed resistance. The results of this analysis helped to guide personalized, precise treatment of the patient despite having two therapeutically adverse events with two recurrences, chromothripsis and mismatch repair (MMR) deficiency. The patient initially had chromothripsis, creating double minutes (DMs) resistant to targeted therapies. To assess the frequency of chromothripsis in GBMs and to emphasize its impact on clinical decisions, we further analyzed the Yale-Glioma cohort composed of 110 whole exome- or whole genome-sequenced tumor-normal pairs. The presented case later acquired MMR deficiency with the second recurrence, leading to resistance to alkylating agent treatments and a hypermutated phenotype. Interestingly, the case displayed a favorable response to immune checkpoint inhibition after acquiring mismatch repair deficiency. With this clinical approach, the patient survived more than 5 years despite the two recurrences (Fig. 1). Our study exemplifies how genomic profiling can successfully guide personalized treatment regimens, even in aggressive cancers such as GBM. Our observations also emphasize the necessity of genomic profiling and comparative analyses for each clinical recurrence or progression.

## Methods

### Ethics and consent of clinical materials

Institutional review board approvals for genetic studies, along with written consent from all study subjects, were obtained at the participating institutions.

### Exome capture and sequencing

Exome capture was performed with a Nimblegen/Roche human solution-capture exome array (Roche Nimblegen, Inc.) [3]. Sequencing of the library was performed on Illumina HiSeq machines (Additional file 1). For molecular profiling of the tumors, we performed deep WES of the primary GBM tumor, first recurrence, and second recurrence, together with the matching normal blood. We achieved high mean target coverage of 209.5×, 229.4×, 199.6×, and 92.6×, respectively. We analyzed all three exome sequencing data sets to detect somatic single-nucleotide variations (SNVs), insertion/deletions (INDELs), copy number variations (CNVs), and structural variations (SVs). We also performed comparative analyses among all three samples to understand the temporal evolution of the tumor under the pressure of not only standard-of-care but also targeted therapies.

For the Yale-Glioma cohort, we achieved mean target coverage of 194.3 and 121.3, for tumors and matching blood, respectively. The average percentage of reads with at least 20× coverage was 91.0 and 88.4% for tumor and blood, respectively.

### Exome sequencing data analysis: somatic SNV/INDEL and CNV analysis

We performed quality control, alignment, PCR duplicate marking, multi-sequence local realignment, base quality score recalibration, and calling of somatic SNV/INDELS (using Haplotyper in Genome Analysis Toolkit, version 2.5) as described previously in [4]. We calculated the clonality rate of mutations based on the variant allele frequency, ploidy at the site, and the admixture rate [5]. We performed the CNV analysis on all tumors using the ExomeCNV package [6]. We used Breakdancer [7] to call breakpoints, applied filtering on the raw calls, and performed annotation using ANNOVAR (Additional file 1).

We used the Mclust package in R (http://www.stat.washington.edu/mclust/) to cluster the unique somatic mutations (coding region and captured non-coding regions) in three tumors based on their clonality rate distributions. Bayesian Information Criteria (BIC) was used to find the model with the optimal number of clusters. The analysis identified clusters, which we used to depict the tumor evolution.

### Whole-genome capture and sequencing

Whole-genome sequencing was performed by Complete Genomics Cancer Sequencing Service v2.0 and downstream analysis was performed with in-house scripts (Additional file 1).

### Tumor cells in culture

Short-term cultured tumor cells were harvested using trypsin, pelleted by centrifugation, re-suspended in a small volume of phosphate-buffered saline (PBS), and incubated for 20 min in a large volume (10–15 ml) of hypotonic 75 mM KCl at 37 °C to increase cell volume and facilitate cell membrane rupture. One volume of 3:1 methanol:acetic acid was slowly added to the cell suspension and cells were pelleted by centrifugation for 5 min at 1200 rpm/400 g. The cell/nuclear pellet was resuspended in 5 ml fresh 3:1 fixative, incubated for 10–15 min at room temperature (RT), and centrifuged again as before. This step was repeated two more times. After the final centrifugation step, the cell pellet was transferred for storage into a 1.5 ml microfuge tube in a small volume of fixative. Unused cells were stored indefinitely in fixative at −20 °C. Prior to spreading on clean slides, cells were resuspended in fresh 3:1 fixative. To obtain cytogenetic preparations/slides with nuclei as flat as possible, the procedure was modified as described in detail elsewhere [8]. Slides were always prepared fresh; only cell pellets were stored long term. After preparation, for fast fixation/dehydration, slides were covered with a long coverslip, ethanol was added to form a thin layer between the slide and the coverslip, and slides were incubated for 1–2 min at 85–90 °C on a heat block, while adding fresh ethanol every few seconds with a pipette in order to prevent complete ethanol evaporation. Afterwards, for tissue “permeabilization”, the dry slides were incubated for 1.5–2 min in a jar with 0.005% pepsin/0.01 M HCl at 37 °C, followed by brief (1–2 min each) rinsing in PBS, 70% ethanol, and 100% ethanol and RT drying. To decrease background signals during FISH, slides were incubated for 10 min with a 0.1 mg/ml solution of RNAse A in PBS, followed by rinsing in PBS, 70 and 100% ethanol (2 min each), and air-dried.

### DNA FISH probe preparation and labeling

We used the following BACs: BAC RPCI-11 433 N15 (for MDM4) and BACs RPCI-11 1112G8, and 148P17 (for EGFR). BAC-containing live bacteria were commercially obtained (Invitrogen). DNA was prepared via mini-preps using the standard procedure (Qiagen miniprep kit). BAC DNA was labeled by nick translation. A 20-μl reaction included: 500 ng BAC DNA, 2 μl 10× *Escherichia coli* buffer, 2 μl 10× DNAseI solution; 1 μl d(ACG), 1 mM each; 0.1 μl dTTP, 5 mM; 0.25 μl DIG-dUTP or BIO-dUTP, 1 mM; 0.5 μl *E. coli* Pol I (10 U/μl; New England Biolabs); and water (to 20 μl). Incubation was for 2 h at 15 °C followed by purification either by ethanol precipitation or using the Qiagen PCR purification kit. The 10× DNAse solution was prepared with 1 μl 1 mg/ml DNaseI (Sigma) + 1 ml water and was always made fresh before use. After purification, the labeled DNA probe was resuspended in 10–20 μl FISH buffer (50% formamide, 2× SSC, 10% dextran sulfate, 1× phosphate buffer = 50 mM 5:1 sodium phosphate dibasic:mono basic, pH 7.0). Cot1 DNA (Invitrogen) was also ethanol precipitated and resuspended at 10 μg/μl in FISH buffer. Prior to FISH experiments, we mixed 4 μl FISH probe with 2–3 μl CotI DNA, placed 6–7 μl per slide, which was covered with a small 12 × 12 mm coverslip and the slide and probe denatured for 3 min at 80–85 °C.

### DNA hybridization and detection

For FISH using simultaneous slide and probe denaturing, 5–6 μl FISH probe was pipetted on the slide, covered with a 12x12mm coverslip, sealed with rubber cement, and both the slide and probe heat-denatured for 3–3.5 min at 80 °C on a heat block, followed by 24-h incubation at 37 °C in a water bath or incubator.

After hybridization, coverslips were removed from the slides with fine forceps. Slides were incubated for 15 min in a jar with 2° SSC at 37 °C, followed by a 15 min incubation in 2× SSC at RT. After a brief rinse in a jar with distilled water, slides were transferred to a jar with 1× PBS. To pre-block the slide, we added 50–100 μl BSDSGS/0.1% Tween (10× BSDSGS contains PBS with 1% bovine serum albumin, 5% donkey serum, 5% goat serum, 0.1% glycine, 0.1% lysine). The primary antibody (mouse-anti-DIG, Sigma) was diluted 1:100 in BSDSGS and 100 μl added to the slide. For BIO-dUTP-labeled probes, at this step we also added Avidin-FITC (or Streptavidin-Alexa 488), 1:100 diluted in BSDSGS/0.1% Tween20. This was followed by a 2 h incubation at 37 °C, though RT incubation works equally well. After a 15-min rinse in PBS, 100 μl of a secondary antibody (usually donkey-anti-mouse-Alexa555, Invitrogen) diluted at 1:500 in BSDSGS/0.1% Tween was placed on the slide and incubated for 15–30 min at RT followed by a 15-min 1× PBS wash. After a brief rinse in distilled water to remove excess salt, the slide was air-dried, mounted with DAPI-antifade (Vector Laboratories), covered with a coverslip, and examined with a microscope (Zeiss Axiophot) using appropriate fluorescence filters. Images were captured with Zeiss software and colored images merged in Photoshop (Adobe).

## Results

**Fig. 2:**
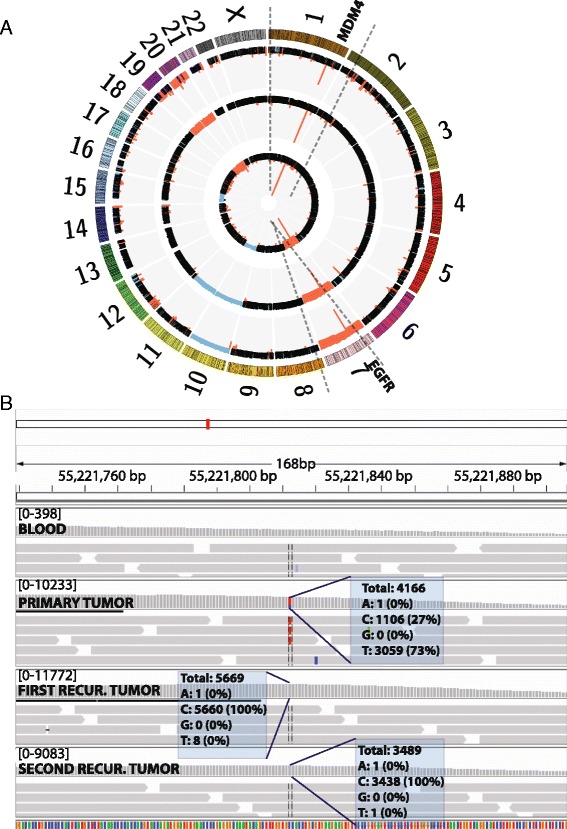
Genomic profile of the presented case. **a** Circos plot representing the CNV status of the original GBM and two recurrences. The *innermost circle* represents the primary tumor, whereas the *middle* and *outer-most circles* depict the first and second recurrences, respectively (*black*, no event; *blue*, deletion; *red*, amplification). **b** IGV plot of the locus of the *EGFR* A289V mutation in three tumors and matching blood. All three tumors show amplification at the locus but only the primary tumor has supporting reads for variant A289V

The patient was a 55-year-old right-handed woman who presented to medical attention in June 2010 after suddenly developing expressive aphasia and confusion. Her initial brain MRI revealed a heterogeneously enhancing infiltrating tumor, and she underwent surgical resection in July 2010. WES analysis of this tumor and matching blood revealed amplification of chromosome 7 and deletion of chromosome 10, together with focal deletion of the cyclin-dependent kinase inhibitor 2A (*CDKN2A*) locus on chromosome 9. Detailed analysis of the CNVs and SVs revealed segments on chromosomes 7p.11 and 1q.32, with more than 20 copies overlapping with the *EGFR* and Mdm2-like P53-binding protein (*MDM4*) genes, respectively (Fig. 2a). Interestingly, regions with high copy numbers also showed an increased number of intra-chromosomal breaks that were supported by high numbers of reads (>100) on WES (Fig. 2a). Taken together, these findings suggested that the tumor cells had undergone chromothripsis [9]. Chromothripsis, shattering, and reassembling of chromosomes leading to amplification and deletion of segments through end joining-based repair or by DM chromosome formation [10] has been observed in 2–3% of cancers [11, 12], including pediatric neuroblastoma [13] and medulloblastoma [14], colorectal cancer, breast cancer [15], melanoma [16], as well as glioma [17].

In addition to high ploidy of *EGFR* in the primary tumor, we also identified an activating ectodomain *EGFR* A289V mutation, which has been previously shown to lead to oncogenic activation [18] and harbor sensitivity to kinase inhibitors, such as lapatinib [19]. The patient was started on standard chemotherapy and radiation with temozolomide and was enrolled in a clinical trial for the receptor tyrosine kinase inhibitor, vandetanib. She completed 12 cycles of adjuvant temozolomide and vandetanib in October 2011 and continued vandetanib alone until disease progression was noted on MRI in February 2013. She underwent a second gross total resection in March, and WES of this recurrent tumor revealed a similar profile to the primary tumor with amplification of chromosome 7 and deletions of chromosome 10 and the *CDKN2A* locus on chromosome 9. Interestingly, when we compared the genomic profiles of the primary tumor and the first recurrence, we observed loss of the tumor cells harboring the activating *EGFR* A289V mutation, most likely due to the targeted anti-*EGFR* therapy with vandetanib, but preservation of *EGFR* amplification (Fig. 2b). This observation suggested that even though the anti-*EGFR* therapy resulted in the eradication of the tumor sub-clone with the activating *EGFR* A289V mutation, it had no impact on the high *EGFR* ploidy. Given these molecular profiling results, which again revealed deletion of the *PTEN* locus, the patient was started on a clinical trial with carboxyamidotriazole orotate (CTO) to target the activated phosphoinositide 3-kinase (PI3K) pathway along with concomitant temozolomide treatment (March 2013). A brain MRI performed 4 months after resection revealed a 4-mm nodular contrast enhancement at the posterior margin of the resection cavity. Of note, this nodule got smaller in subsequent scans (data not shown).

**Fig. 3:**
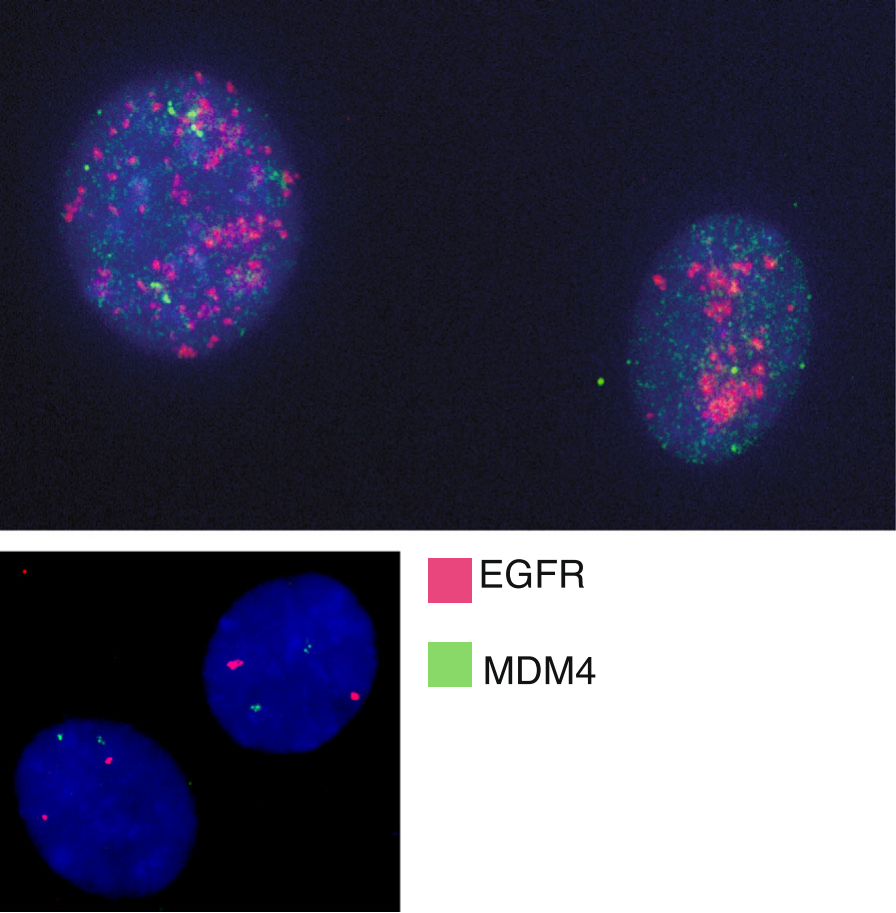
FISH analysis for *EGFR* and *MDM4*. FISH analysis of the second recurrence tumor displays the high ploidy states of *EGFR* (*red*) and *MDM4* (*green*). The smaller panel displays a control sample with two copies of *EGFR* and *MDM4*

During the combination therapy of CTO and temozolomide, a second recurrence, diagnosed based on both clinical and radiographic evidence, occurred in June 2014. At that time, the patient developed worsening speech and a new area of nodular contrast enhancement along the posterior and inferior margins of the resection cavity. Based on these findings, she underwent a third resection in June 2014. WES of this second tumor recurrence again showed chromosome 10 and *CDKN2A* deletion but even more interestingly still high ploidy of the *EGFR*/*MDM4* loci (>10 and >6, respectively). Given this observation, we tested whether the persisting high *EGFR* ploidy was due to the formation of DMs or homogeneously staining regions, which are extra-chromosomal and intra-chromosomal amplifications of segments, respectively. Indeed, *EGFR* and *MDM4* FISH analysis of the patient-derived primary GBM cells from the second recurrence showed the presence of DMs, which are known to be resistant to targeted chemotherapies as previously reported (Fig. 3) [20, 21].

After demonstrating chromothripsis affecting also the second recurrence, we focused on the somatic mutation count of the second recurrence tumor. This tumor harbored a hypermutated phenotype (2079 somatic coding mutations versus 68 and 70 in the primary and first recurrence tumors, respectively). Further analysis revealed a deleterious missense mutation affecting the MutS domain III (T767I) of mutS homolog 6 (*MSH6*), a gene involved in the DNA MMR mechanism, which was shown to lead to hypermutated cancers [22, 23].

Based on the results supporting formation of DMs as well as the hypermutated phenotype, a combination therapy targeting both of the molecular events was designed. The patient was started on hydroxyurea and an immune checkpoint inhibitor, pembrolizumab, targeting the PD-1 molecule, together with radiation therapy, potentially helping to release the immune targets. Indeed, recent studies reported other hypermutated solid tumors, including colorectal, endometrial, gastric, and small bowel cancers, as well as cholangiocarcinoma, to be potentially susceptible to immune checkpoint inhibitors [24].

Remarkably, in March 2015, 5 months after the start of the combination therapy of pembrolizumab and hydroxurea in October 2014, an MRI revealed a decrease in tumor size. The disease remained stable without further progression until mid-June 2015, at which time a repeat scan revealed increased perfusion, suggesting progression with leptomeningeal spread. Hydroxyurea was stopped and bevacizumab was started (Fig. 1). After being clinically stable for several months, her neurological condition deteriorated and she died in November 2015.

**Fig. 4:**
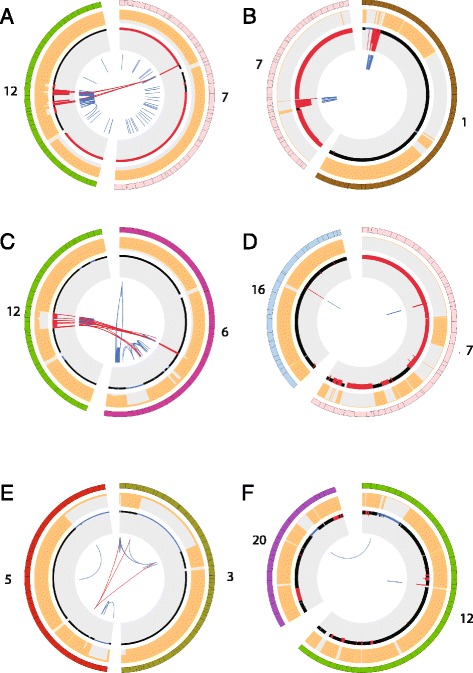
Cases with chromothripsis in the Yale-Glioma cohort. Circos plots of six GBM cases with chromothripsis (only affected chromosomes are plotted). The *outer-most circles* depict the chromosomes (numbers shown) and shift in lesser allele frequency (in *orange*), respectively. The next track plots the copy number status (*black*, no event; *blue*, deletion; *red*, amplification). Links in the *inner track* display the inter- or intra-chromosomal breaks. **a** A GBM with chromothripsis affecting chromosome (chr) 12 and chr7 with high level amplification and a large number of inter-chromosomal breaks. **b** Chr1–chr7 chromothripsis event with high level amplification. **c** Chr12–chr6 event with high level amplification and inter-chromosomal breaks. **d** Chr7–chr16 chromothripsis with high level of amplification. **e** Chr3–chr5 event causing deletion in secondary GBM case. **f** Chr12–chr20 deletion with inter-chromosomal breaks

Given the potential clinical implications of these molecular findings, specifically the chromothripsis and hypermutated phenotypes, we next interrogated the Yale-Glioma cohort for similar events. This collection contains 110 tumor-normal matched primary or secondary gliomas with WES data, 24 of which have also been whole-genome sequenced. We found that 31% of all primary GBMs (16/52) and only 1.7% (1/57) of secondary GBMs had undergone chromothripsis (Fig. 4; Additional file 2: Figure S1). Besides the frequently altered, previously reported loci (chromosomes 7p11 and 12q13-15) [25], our analysis also revealed novel loci on chromosomes 1p36 (harboring *MTOR*, *n* = 1), 1q32 (harboring *MDM4*, *n* = 2), 6q21 (harboring the autophagy protein 5 gene, *ATG5*, *n* = 1) and 16q13 (harboring a cluster of metallothionein (*MT*) genes, *n* = 1) to be amplified in GBM samples (Fig. 4a–d; Additional file 2: Figures S2 and S3). Among these, *ATG5* is responsible for autophagasome formation and, to our knowledge, this is the first time *ATG5* is reported to have high copy number amplification due to chromothripsis. We correlated the observed locus ploidy with the available gene expression data to show that the high *ATG5* ploidy was concurrent with increased gene expression, suggesting mechanistic significance (Additional file 2: Figure S2). *MT* genes are known to bind to physiological or xenobiotic heavy metals, such as alkylating agents, used therapeutically, which leads to resistance and treatment failures [26]. In addition, we identified two samples with inter-chromosomal breaks linking deleted segments; one secondary GBM case between chromosomes 3p24-p13 and 5p13.2 (Fig. 4e) and another primary GBM case between chromosomes 12p13 and 20q13.12-13 (Fig. 4f). Further studies are required to assess the frequency and clinical significance of such events. We also checked the Yale-Glioma cohort for any additional hypermutated cases and identified a single patient (out of 52 primary GBMs, 1.9%) to have a MMR deficiency leading to a hypermutated phenotype. This tumor also harbored a somatic, predicted deleterious missense mutation located on the MutS domain II of *MSH6* (C687Y).

**Fig. 5:**
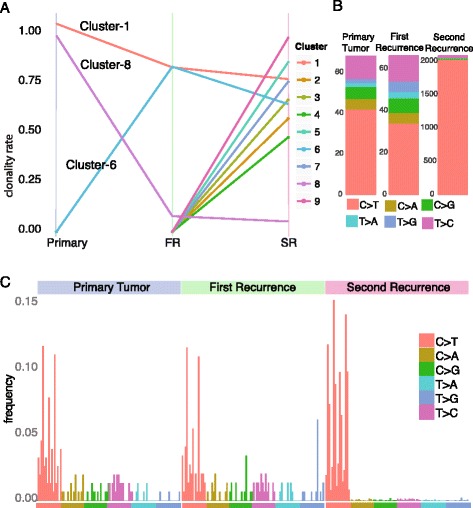
Clonal evolution and mutation signature analysis of the presented case. **a** Clonal evolution of the primary tumor, first recurrence and second recurrence. Nine unique somatic mutation clusters are identified. Whereas cluster 1 contains the high clonal somatic mutations that are preserved through the evolution of the tumor, cluster 8 represents mutations that are lost (or preserved with very low clonality) during recurrence. Interestingly, all the remaining clusters contain mutations that are unique to the second recurrence tumor, with the exception of mutations in cluster 6, which emerged during the first recurrence. **b** Mutational signatures of tumors reveal an increased burden of C to T alteration for the second recurrence due to acquired MMR deficiency. All mutational signatures are shown with the color codes explained at the bottom. **c** Distribution of somatic mutation signatures including the 5′ and 3′ flanking bases. The second recurrence has an increase in C > T alterations in addition to the hypermutated phenotype and displays a signature similar to the one induced by the alkylating agents [33]

We later performed a longitudinal analysis of our patient to assess the temporal evolution of the tumor and potential impact of the acquired MMR deficiency. We performed a model-based clustering analysis of the clonality rates of the unique somatic mutations, including both the coding and non-coding (captured) ones in all three samples (Additional file 3). This analysis revealed that while one major clone was preserved across all tumors (cluster 1), another clone was lost (cluster 8, including *EGFR* A289V) and many new sub-clones emerged in the second recurrence, consistent with the acquired MMR deficiency (Fig. 5a). In addition, the second recurrence displayed a distinct mutation signature, with a drastic increase in the C > T transition ratio (97 versus 68 and 54% in the second recurrence versus the primary tumor and first recurrence, respectively), consistent with the previously described findings after exposure to alkylating agents (Fig. 5b, c) [27–30].

## Discussion

The longitudinal genomic profiling carried out in this study demonstrates that the genomic profile of a tumor can evolve with treatments, leading to selection of resistant sub-clones while eradicating others. Our observations also emphasize the necessity of genomic profiling and comparative analyses for each clinical recurrence or progression. We demonstrate that intra-tumoral heterogeneity in GBM is caused by temporal evolution of the tumor as well as mechanisms leading to large-scale genomic alterations, such as chromothripsis, creating therapy-resistant clones. Moreover, we report chromothripsis events leading to DMs to be a frequent event in primary GBMs, especially when compared to other cancer types. We also identified novel loci being affected by chromothripsis by extending our study to the Yale-Glioma cohort, which might have effects on the targeted treatments. Hence, the presence of DMs, which would limit the therapeutic success of targeted therapies, should be strongly considered when personalized glioma treatments are planned, such as hydroxurea or gemcitabine [31, 32]. The new loci presented in this study to be affected by chromothripsis should be further investigated to access the functional and clinical significance. Finally, we presented the potential positive response to checkpoint inhibitors in gliomas, where the cases present resistance to alkylating agent treatment due to acquired MMR deficiency during progression. Further studies will be needed to assess the exact extent of the therapeutic impact of the immune checkpoint inhibitors in the treatment of gliomas with hypermutated phenotypes.

## Conclusions

Our study exemplifies how genomic profiling can successfully guide personalized treatment regimens, even in aggressive cancers such as GBM. Our study also demonstrates that intra-tumoral heterogeneity, one of the causes of therapy resistance in GBMs, does not occur due just to the variation in somatic alterations but also to mechanisms leading to large-scale genomic alterations, such as chromothripsis. Moreover, our study presents the checkpoint inhibitors as a new potential targeted treatment agent in gliomas, especially in cases with acquired MMR deficiency resulting in a hypermutated phenotype and resistance to standard alkylating agent treatment.

Overall, with the presented case, we demonstrate the importance of longitudinal genomic profiling to adjust to the dynamic nature of treatment-induced molecular changes to improve the outcomes of precision therapies.

## Additional files

Additional file 1: Supplementary methods. (PDF 90 kb)
Additional file 2: Supplementary figures. **Figure S1** Circos plots of cases in the Yale-Glioma cohort that are identified to have gone through chromothripsis. **Figure S2** ATG5 gene amplification through chromothripsis. **Figure S3** Increased levels of MT group genes by a focal amplification in a whole-genome genotyping experiment. (PDF 465 kb)
Additional file 3: Supplementary table. Somatic mutations in three tumors of the presented case. (XLSX 798 kb)

## Abbreviations

CNV: Copy number variation
CTO: carboxyamidotriazole orotate
DM: Double-minute
GBM: Glioblastoma multiforme
INDEL: Insertion/deletion
MMR: Mismatch repair
RT: Room temperature
SNV: Single-nucleotide variation
SV: Structural variation
WES: Whole-exome sequencing
