# Supplementary material for: Longitudinal analysis of treatment-induced genomic alterations in gliomas
Source: Genome Med. 2017 Feb 2;9:12. doi: 10.1186/s13073-017-0401-9 (PMC5290635; doi:10.1186/s13073-017-0401-9)
Supplement: Additional file 1: — Supplementary methods. (PDF 90 kb) [file 13073_2017_401_MOESM1_ESM.pdf]

Supplementary Methods

Exome Capture and Sequencing:

Nimblegen/Roche human solution-capture exome array (Roche Nimblegen, Inc.) was used to capture the exomes of blood and tumor samples according to the manufacturer's protocol with modifications (1). Sequencing of the library was performed on Illumina HiSeq instruments using 74 base pairs paired-end reads by multiplexing two tumor samples or three blood samples per lane.

We have performed a deeper coverage of tumors as compared to matching blood samples (average target coverage was 194.3 and 121.3, respectively). The average percentage of reads with at least 20x coverage was 91.0% and 88.4% for tumor and blood, respectively .

We have performed quality control steps on raw Illumina reads, followed by alignment using BWA and STAMPY, PCR duplicate marking together with alignment metric calculation by Picard, multi sequence local realignment and base quality score recalibration by GATK as described previously in (2).

Somatic SNP/INDEL and CNV Analysis:

Haplotype caller implemented in Genome Analysis Toolkit (GATK, version 2.5)(3) was used to call variants in tumor-normal pairs. Later we calculated a somatic score according to the method described by Li(4). Variant annotation was performed using Variant Effect Predictor and filtering was performed as detailed in (2).

We performed the CNV analysis on all tumors using the ExomeCNV package.

Exome SequenSomatic Structural Variation Analysis:

We have used Breakdancer (5) to call breakpoints. We have used the multi-sample calling mode to assess the somatic status of breakpoints. We have filtered intra-chromosomal breakpoints if: (i) breakpoints were less than 200 bp apart, (ii) there were any supporting reads from the matching blood, (iii) there were less than 8 supporting reads in the tumor, or (iv) breakpoints had a quality score of less than 40. We later annotated the breakpoints using ANNOVAR for hg19 refSeq genes, cyto-band information, segmental duplications, repeat masked regions. Based on these annotations we later filtered breakpoints that overlap with segmental duplications or any repeat elements. We have excluded the breakpoints with supporting reads less than 20 in the Circos plots.

Mutation Signature Analysis:

We have calculated the mutation signature of individual tumors' somatic mutations by considering 6 major mutation classes, i.e., G:C > T:A, G:C > A:T, G:C > C:G, A:T > G:C, A:T > C:G, A:T > T:A. We also calculated the mutation signature using the 5' and 3' based flanking the variation, which leads to 96 classes of signatures.

Clonality Analysis:

Clonality rate is defined to be the percent of tumor cells harboring the identified somatic mutation and correlates with the temporal evolution of the tumor (6, 7).

We estimated the percent of cells that harbor the heterozygous somatic mutations based on the observed variant allele frequency, ploidy at the site of variant and the admixture rate similarly as previously described (8).

We later used the Mclust package in R to cluster the unique somatic mutations (coding region and captured non-coding regions) in 3 tumors based on their clonality rate distributions. Bayesian Information Criteria (BIC) was used to find the model with optimal number of clusters (http://www.stat.washington.edu/mclust/; Fraley and Raftery, 2002 and Fraley and Raftery 2006). The analysis identified clusters, which we used to depict the evolution of tumor.

Whole Genome Capture and Sequencing:

Whole genome sequencing was performed by Complete Genomics Cancer Sequencing Service v2.0 and downstream analysis was performed with in house scripts. An initial fast alignment was performed on (GRCh37) reference genome followed by local *de novo* assembly for regions candidate for variation as detailed in (9). Somatic small alterations and CNVs were reported by Complete Genomics (10). Non-diploid model for the Somatic CNV calls were used from the report, as gliomas can represent gross copy number alterations across the genome. The segments of varying coverage levels were used in Circos plots instead of 100kb merged segments, as focal alterations are critical.

Downstream filtering, integration and analysis scripts were developed in-house. Somatic structural breakpoints reported in the high confidence dataset by Complete Genomics were further filtered out if (i) frequency in the baseline control set was >

0, (ii) the breakpoints were overlapping with segmental duplications, or (iii) number of supporting reads in tumor sample was < 20.

References:

1. Bilguvar K, Ozturk AK, Louvi A, et al. Whole-exome sequencing identifies recessive WDR62 mutations in severe brain malformations. *Nature.* 2010;467(7312):207-10.
2. Erson-Omay EZ, Çağlayan AO, Schultz N, et al. Somatic POLE mutations cause an ultramutated giant cell high-grade glioma subtype with better prognosis. *Neuro-Oncology.* 2015.
3. DePristo MA, Banks E, Poplin R, et al. A framework for variation discovery and genotyping using next-generation DNA sequencing data. *Nat Genet.* 2011;43(5):491-8.
4. Li H. A statistical framework for SNP calling, mutation discovery, association mapping and population genetical parameter estimation from sequencing data. *Bioinformatics.* 2011;27(21):2987-93.
5. Chen K, Wallis JW, McLellan MD, et al. BreakDancer: an algorithm for high-resolution mapping of genomic structural variation. *Nat Meth.* 2009;6(9):677-81.
6. Nik-Zainal S, Van†Loo P, Wedge DC, et al. The Life History of 21 Breast Cancers. *Cell.* 2012;149(5):994-1007.

7. Yates LR, and Campbell PJ. Evolution of the cancer genome. *Nat Rev Genet.* 2012;13(11):795-806.

8. Stephens PJ, Tarpey PS, Davies H, et al. The landscape of cancer genes and mutational processes in breast cancer. *Nature.* 2012;486(7403):400-4.

9. Drmanac R, Sparks AB, Callow MJ, et al. Human Genome Sequencing Using Unchained Base Reads on Self-Assembling DNA Nanoarrays. *Science.* 2010;327(5961):78-81.

10. Carnevali P, Baccash J, Halpern AL, et al. Computational Techniques for Human Genome Resequencing Using Mated Gapped Reads. *Journal of Computational Biology.* 2011;19(3):279-92.
