# Supplementary material for: Longitudinal analysis of treatment-induced genomic alterations in gliomas
Source: Genome Med. 2017 Feb 2;9:12. doi: 10.1186/s13073-017-0401-9 (PMC5290635; doi:10.1186/s13073-017-0401-9)
Supplement: Additional file 2: — Supplementary figures. Figure S1 Circos plots of cases in the Yale-Glioma cohort that are identified to have gone through chromothripsis. Figure S2 ATG5 gene amplification through chromothripsis. Figure S3 Increased levels of MT group genes by a focal amplification in a whole-genome genotyping experiment. (PDF 465 kb) [file 13073_2017_401_MOESM2_ESM.pdf]

Supplementary Figures

Figure S1

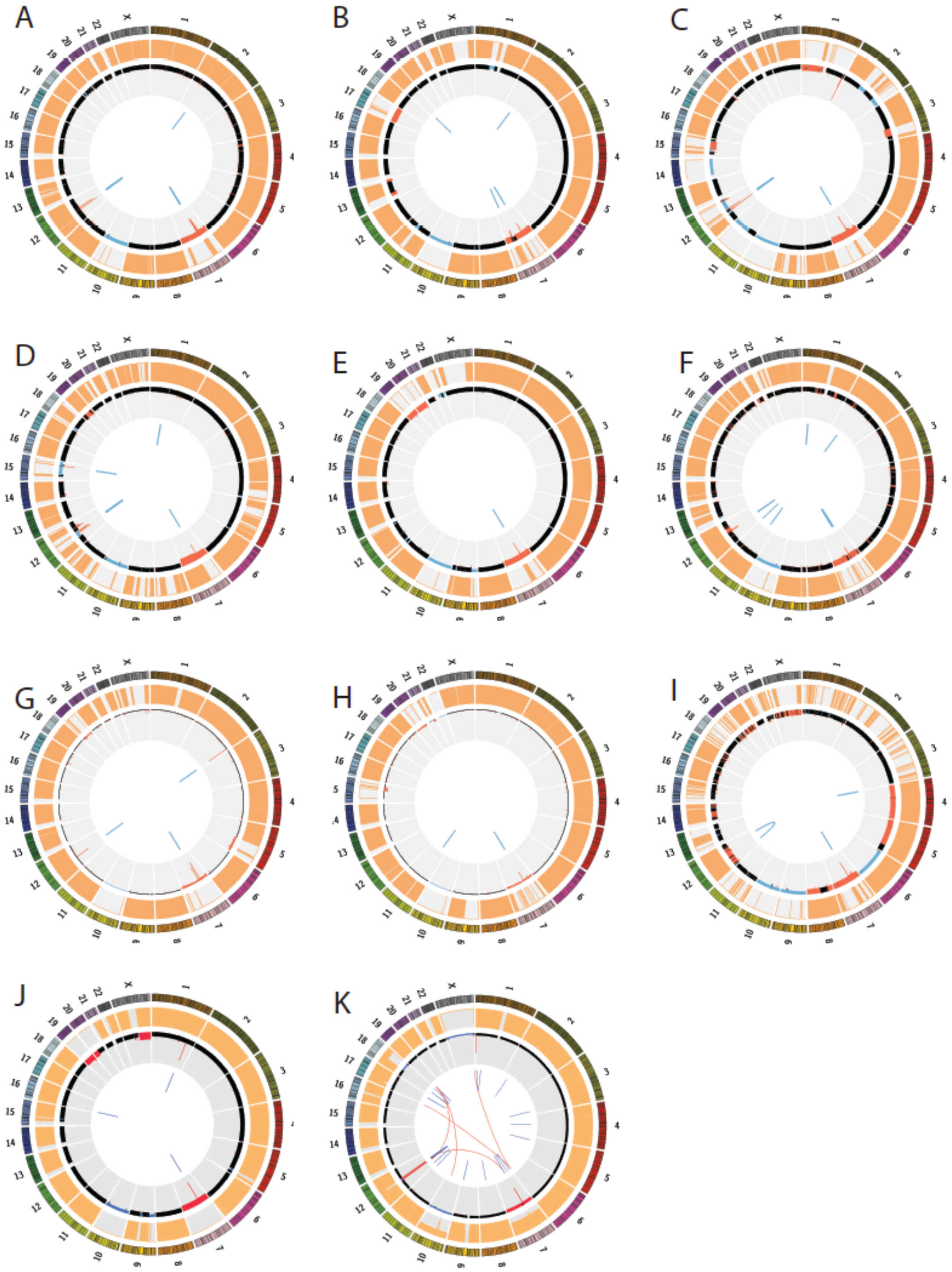

Figure S1: Circos plots of cases in Yale-Glioma cohort that are identified to have gone through chromothripsis. Out-most circle after the chromosome tracks, depicts the shift in lesser-allele-frequency in orange. The second track plots the copy number status, black: no-event, blue: deletion, red: amplification. Links in the inner track display the inter/intral chromosomal links.

Figure S2

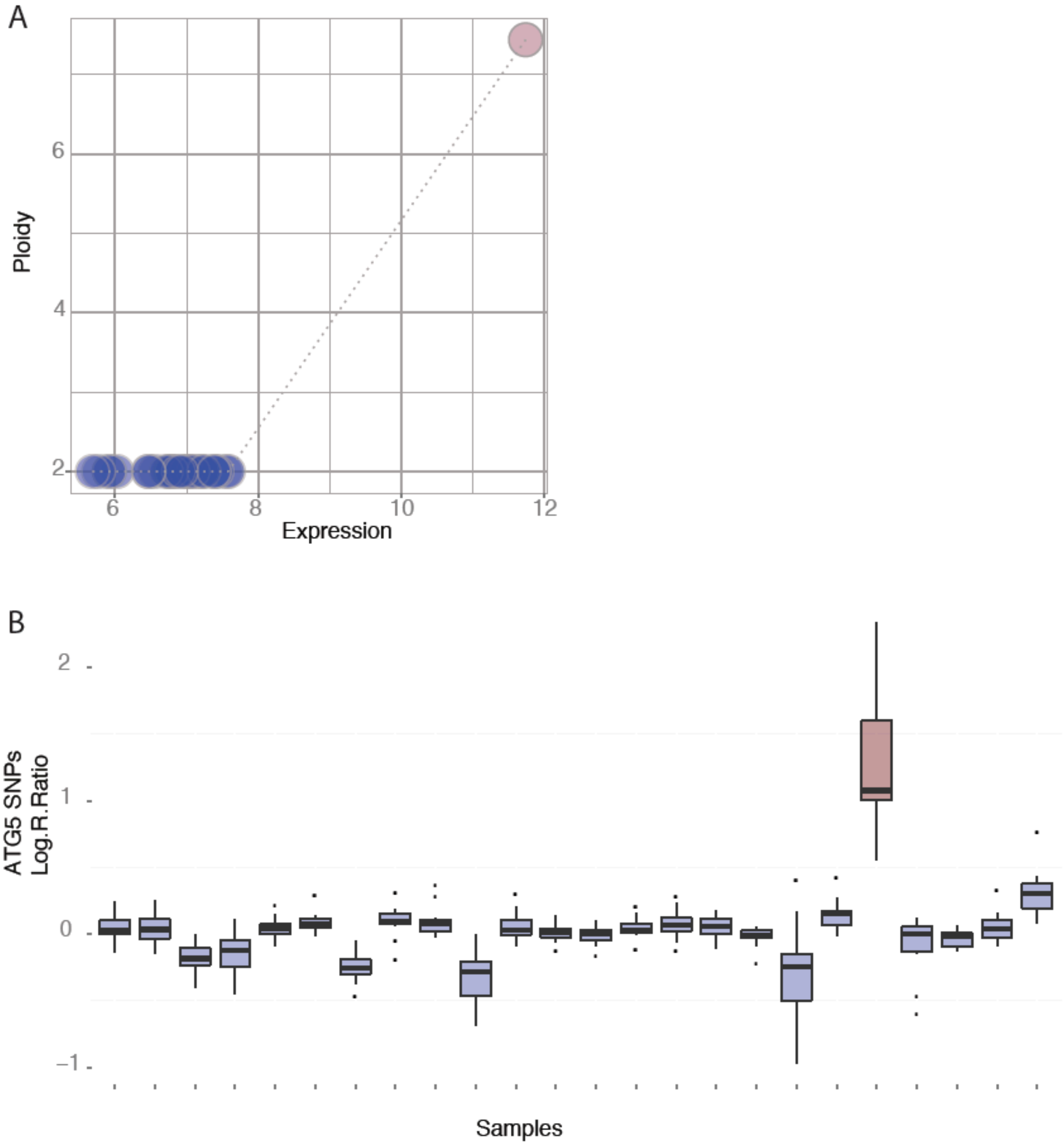

Figure S2: ATG5 gene amplification through chromothripsis.(A) Correlation of gene expression and copy number state for ATG5 gene. The sample with putative double-minute with ATG5, caused by chromothripsis between chromosome 12 and chromosome 6, (shown in pink) displays high-ploidy correlating with high levels of expression. (B) Confirmation of WEX-ploidy estimation using whole genome genotyping data. The sample with putative DM carrying ATG5 (shown in pink) has the highest log.r ratio for SNPs overlapping with ATG5 gene.

Figure S3

A

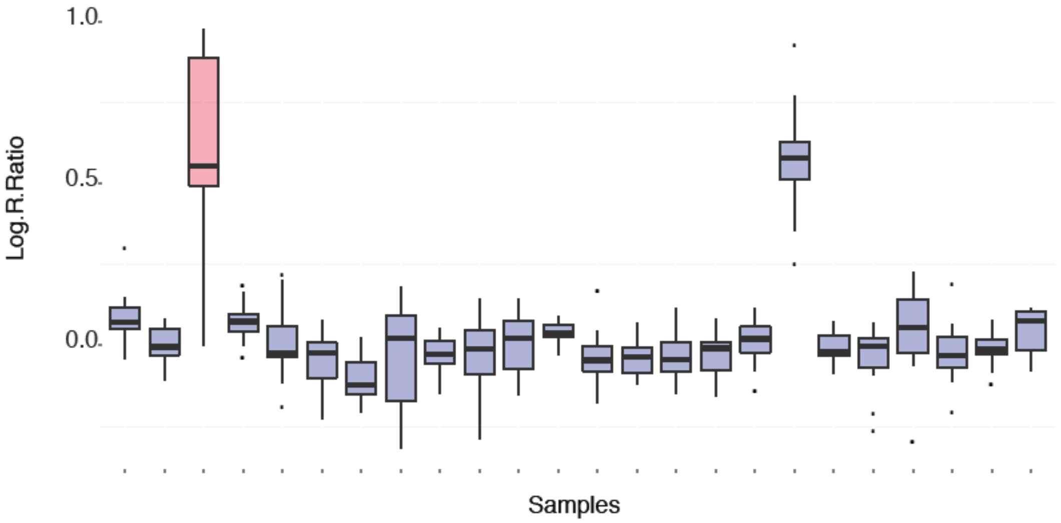

B

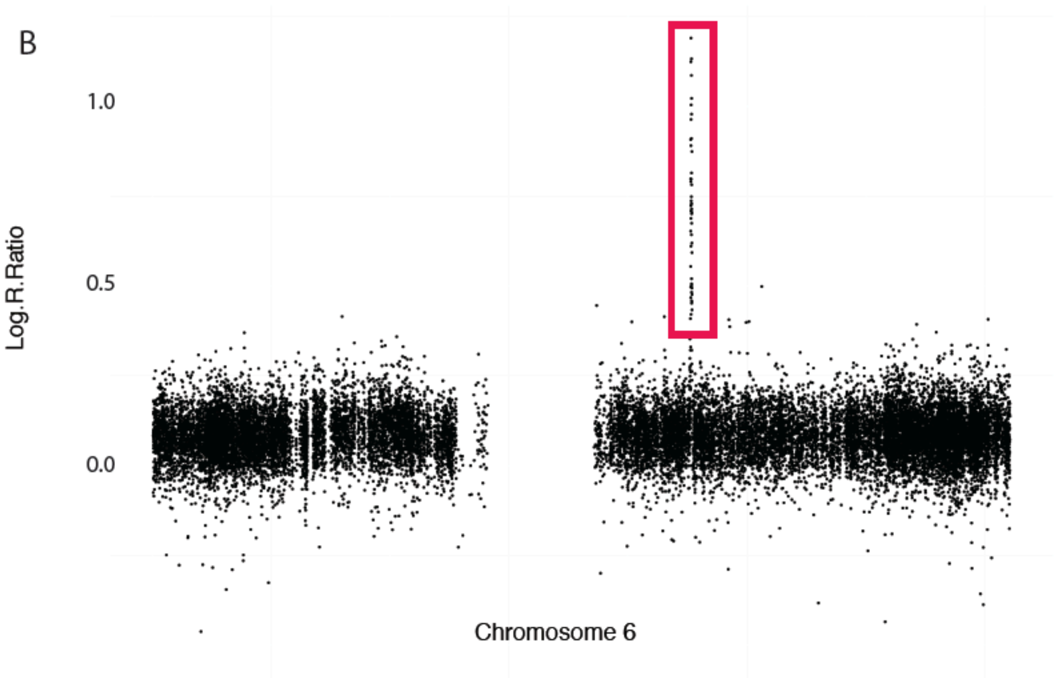

Figure S3: **Increased levels of MT group genes by a focal amplification in whole genome genotyping experiment.** (A) Sample wise Log.R.ratio levels for SNPs overlapping with MT genes on chr16q13 depicting increased values for the sample with putative DMs of MT genes, shown in pink. (B) All SNPs' Log.R.Ratio levels in chr16q13 region, showing focal increase in the segment overlapping with MT genes in single sample with putative DMs of MT genes.
